# Assessing the effects of design modifications on the use of wildlife exits designed for endangered Texas ocelots

**DOI:** 10.1371/journal.pone.0323705

**Published:** 2025-06-24

**Authors:** Rupesh Maharjan, Jamie E. Langbein, John H. Young, Kevin Ryer, Alejandro Fierro-Cabo, Md Saydur Rahman, Richard J. Kline

**Affiliations:** 1 School of Earth, Environmental, and Marine Sciences, University of Texas Rio Grande Valley, Brownsville, Texas, United States of America; 2 Texas Department of Transportation, Environmental Affairs Division, Austin, Texas, United States of America; 3 School of Integrative, Biological, and Chemical Sciences, University of Texas Rio Grande Valley, Brownsville, Texas, United States of America; University of Brighton, UNITED KINGDOM OF GREAT BRITAIN AND NORTHERN IRELAND

## Abstract

Roadside exclusionary fencing is commonly used on highways to prevent wildlife-vehicle collisions. Although it can mitigate wildlife road mortality by limiting their access to the road, it can also create a barrier for wildlife stranded within the right-of-way. On State Highway 100 in Texas, the Texas Department of Transportation installed 10 wildlife exits (WEs) to allow endangered ocelots and other wildlife to escape the fenced roadway and minimize wildlife-vehicle collisions. Our study compared three types of WE designs within the same area from 2019 to 2024 to assess their effectiveness. The first design (Design A) was without a door and berm, and the second design (Design B) had a raised berm (10 WE sites) with a door (six WE sites) and no door (four WE sites). Lastly, a third design (Design C) had all the structural features of Design B, except for the raised berm removed from all ten WE sites. We used the approaches of four meso-carnivore target species (coyote, bobcat, northern raccoon, and striped skunk) as a metric and binomial generalized linear model as a statistical method to evaluate the effectiveness between three designs. The statistical analysis showed that the raised berm in Design B was the major cause for a decline in the approaches of meso-carnivore communities toward the WE sites. With the berm removal in Design C, the approaches of target species from road to habitat increased significantly in the sites without a door. Our study indicates that the WE door design might be another structural cause for limiting its effectiveness, where significantly lower approaches were recorded for meso-carnivores in the sites with a door compared to those without a door in Design C.

## Introduction

Roads and traffic have deleterious impacts at the individual, population, and community levels for wildlife. The construction of road networks and their expansion result in the clearing of vegetation, which causes habitat loss along and adjacent to the roads [1]. Moreover, roads act as a barrier that can impede wildlife movement, and reduce dispersal and gene flow, which could hinder populations’ survival [2]. However, some species show avoidance to the road-effect zone, an area where the ecological effect of the road impacts its adjacent landscape, due to high traffic disturbance and habitat degradation while some reptiles (bask on the warm surface of the road) and herbivores (forage on the roadside vegetation) are attracted to roads, increasing the species vulnerability to wildlife-vehicle collision [3,4].

Wildlife-vehicle collision (WVC) is a major threat to both humans and wildlife globally. It contributes to a decline in global biodiversity due to non-natural mortality, which can lead to the extinction of threatened species [5]. Wilkins et al. [6] mentioned that around 5% of vehicle accidents each year in the U.S. are caused by WVCs. Landscape bridges, wildlife underpasses, overpasses, and modified culverts are some of the most common forms of wildlife crossing structures (WCSs) designed and used to mitigate the negative impact of roads [7]. These structures are designed to facilitate safe wildlife crossings over roads, however, they cannot be installed along the entire length of the road [8]. Thus, exclusionary fencing is often used to prevent wildlife from accessing roads and to guide them toward crossing structures. Although fences are effective in preventing wildlife from entering the roads, they are not always practical, particularly for large roads with several entry points or fence gaps. Additionally, the behaviors (jumping, climbing, and digging) of species make it even harder to keep them off the roadway [9]. This led to the development of various wildlife escape structures like one-way doors [10], jump-outs [7,11], and wildlife exits [12].

The earliest study on one of the escape designs, conducted by Reed et al. [13] along Interstate Highway 70 in Colorado, investigated the effectiveness of one-way gates. This design consists of a flap or barrier that opens outward, allowing wildlife to pass through in one direction while remaining closed to prevent their return. Although these gates are designed only to be used in one direction, animals have been reported to be using them from both directions [9,12]. Reed et al. [13] found that large species like elk (*Cervus canadensis*) and moose (*Alces alces*) can even bend and transform the shape of tines, an elongated metal part of the one-way door that helps guide or control the movement of wildlife, making it into a two-way opening. Later, other designs of wildlife escapes such as wildlife jump-outs, also known as earthen escape ramps, were implemented whose success rate was comparatively higher than one-way gates [10,14]. These escape structures have earthen mound, angled and leveled up to the height of exclusion fence, then drop off abruptly towards the habitat side of the fence [10]. The structures were designed to encourage wildlife to walk up the earthen ramp and then escape or jump out towards the habitat side across the fence [15].

The effectiveness of these escape measures can be complex when dealing with multiple wildlife species. This structure might be effective for some species such as bears (*Ursus* spp.), coyotes (*Canis latrans*), and mountain lions (*Puma concolor*) [11,15], but not for other species such as white-tailed deer (*Odocoileus virginianus*) [9,10]. So, it is crucial to come up with species-specific designs. One of the species whose population has been severely affected by habitat fragmentation due to the roads and WVCs is the ocelot (*Leopardus pardalis*). This elusive medium-sized feline species is one of the most endangered species in the United States whose habitat is mainly confined to the southern regions of Texas [16,17]. In addition, the existence of high-speed roadways in this part of Texas makes it harder for wildlife species to cross the road leading to an increase in WVCs. Over the past decade, ten ocelots have died from vehicle collisions, with three of these fatalities attributed to vehicle collisions between 2010 and 2014 at three different sections of State Highway 100 (SH 100). Following the deaths of ocelots from vehicle collision accidents, the Texas Department of Transportation (TxDOT) in consultation with the US Fish and Wildlife Service (USFWS), constructed five new or improved WCSs along this section of the roadway.

**Fig 1:**
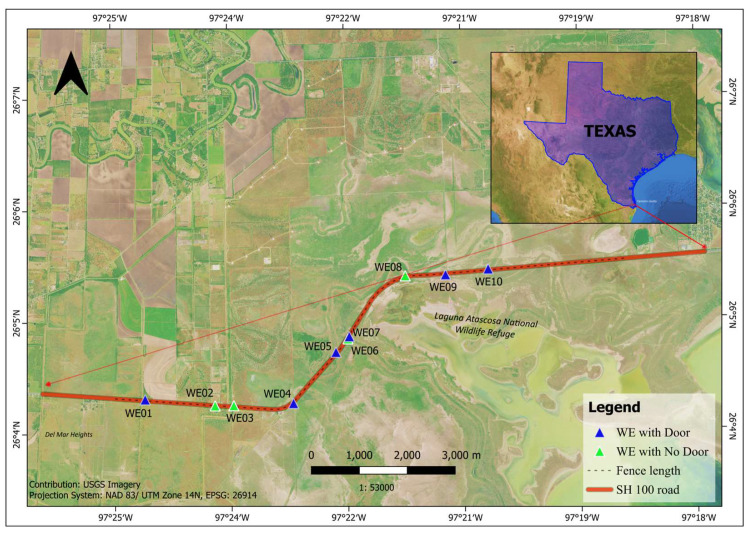
Location of wildlife exits along the section of State Highway 100. The Texas Department of Transportation constructed ten wildlife exits along 11.9 km section of fenced roadway starting from Laguna Vista to Los Fresnos in South Texas. The wildlife exits were installed to allow wildlife to escape the right-of-way and prevent wildlife vehicle collisions.

To deter wildlife from crossing the highway and guide them to WCSs, TxDOT installed a 1.7 m tall continuous chain-link fence made of Geo-mesh polypropylene fiber (GEO 55) along the section of SH100. Initially, TxDOT’s focus was on preventing ocelots and other wildlife from entering the roadway and providing a safer crossing under the highway using WCSs. However, it was observed that some wildlife still entered the right-of-way of SH100 through the wildlife guards and fence gaps [12]. Since there weren’t any existing escape structures along the fenced section of SH 100, wildlife were trapped inside the right of way (ROW). To address this issue, TxDOT installed 10 wildlife exits (WEs) at different sites of the fenced roadway (Fig 1).

This study emphasized four target species (meso-carnivores: coyote – *Canis latrans*, bobcat – *Lynx rufus*, northern raccoon – *Procyon lotor*, and striped skunk – *Mephitis mephitis*) because the concept of WEs was primarily implemented for the ocelot to exit the ROW. Therefore, the WE size did not enable its use by larger species such as white-tailed deer (*Odocoileus virginianus*), javelina (*Tayassu tajacu)*, feral hog (*Sus scrofa)*, and nilgai (*Boselaphus tragocamelus)*. The overall purpose of this research was to compare the effectiveness between three different WE designs using the approaches of meso-carnivores towards WEs from the road to the habitat side of the fence. To assess the effectiveness, the following hypotheses were tested: (i) approaches of meso-carnivores significantly increase in Design C compared to Design A, and (ii) raised berm removal in WE sites significantly increases the approaches of the target species. We tested this hypothesis assuming that the raised berm in Design B was the major structural factor reducing meso-carnivores approaches to WEs and that, over time, meso-carnivores would start using those WEs more frequently.

## Materials and methods

### Study area

In 2006, the Texas Department of Transportation (TxDOT) installed concrete traffic barriers to reduce vehicle collisions along the 11.9 km section of State Highway 100 (SH100), extending from Laguna Vista to Los Fresnos in South Texas (Fig 1). A continuous chain-link fence with GEO 55 was also installed to prevent wildlife from accessing the roadway and guide them towards the crossing structures. The study area borders the Laguna Atascosa National Wildlife Refuge, which is a critical habitat for one of the last remaining ocelot populations in South Texas. The area comprises wide grassland, Tamaulipan thornscrub vegetation, and Gulf coastal prairie habitat. The temperatures reach an average of 35^o^C in the summer, to an average of 12^o^C in winter [18]. The area has lands that are managed by US Fish and Wildlife Service while others are privately owned agriculture fields and ranchlands. The study area is home to diverse wildlife communities that are unique to South Texas, including the federally endangered ocelot and Aplomado falcon (*Falco femoralis*) along with the state-threatened Texas tortoise (*Gopherus berlandieri*).

### Wildlife exit designs

**Fig 2:**
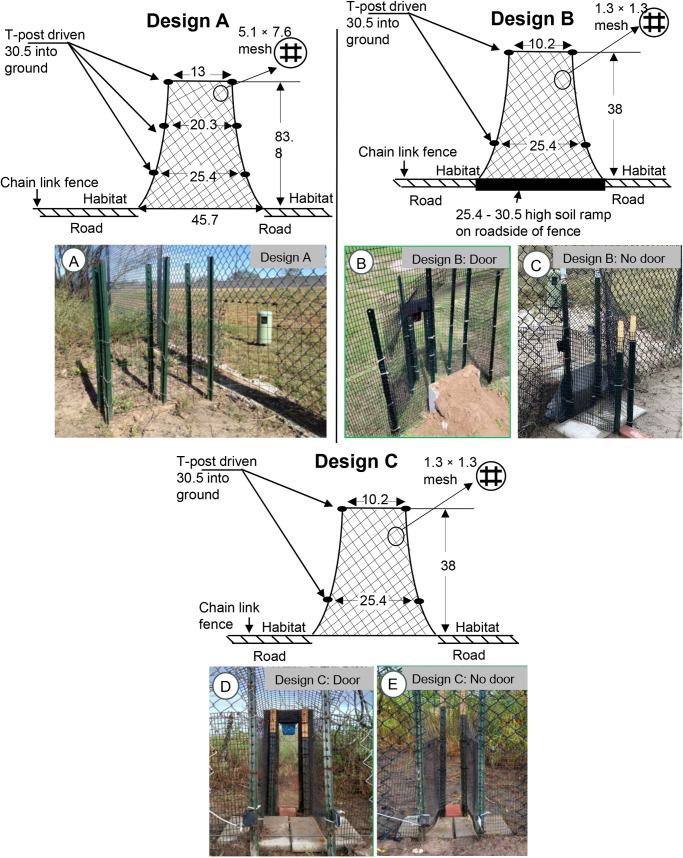
Three wildlife exit (WE) designs installed along the fenced roadway of State Highway 100, Cameron County, Texas between 2019 to 2024. (A) Design A was installed in 2019 to allow small-medium sized wild animals to escape from the right-of-way. This design didn’t have any barriers or doors that allowed wildlife to easily access the roadway. (B, C) Design B installed in 2022 as a replacement for Design A to prevent wildlife from accessing the roadway where raised berms were installed at all 10 WE sites with six sites having a one-way door and the remaining four without a door. (D, E) Design C installed in 2023 where all raised berms were removed, keeping door and structural features same as in Design B. All measurements are in cm.

Three different types of WE designs were tested between 2019 and 2024 to compare their effectiveness. The first Design (referred to as Design A onwards, Fig 2A) had a simple structural design installed at 10 sites, without any doors and barriers [12]. The second design (Design B) had a raised berm (Fig 2B, 2C) placed at the entrance of all 10 WE sites to elevate the entrance height facing the ROW. Six of those WEs featured transparent one-way plastic door and four without the door. The third design (Design C, Fig 2D, 2E) had a raised berm removed from all 10 WE sites (six with door and four without door) keeping other structural features the same as in Design B.

WEs were designed for the endangered ocelots, but their dimensions permit use by a wide range of medium to small-sized wild animals residing within the study area. All WEs (A, B, and C) varied from each other in terms of their structural design. WE in Design A were constructed to create a funnel with six T-posts of a height of about 1.2 m, which was driven 30.5 cm deep in the ground. This structure was covered with 16-gauge black PVC-coated welded wire having a 5.1 × 7.6 cm mesh panel and tightened with hog rings with the chain-link fence. The design was 61 cm tall and 45.7 cm wide, funneled through the fence, and narrowed to 13 cm before reaching the final opening at the habitat side with the length extending to 83.8 cm (Fig 2A).

The opening of all WEs in Design B was reinforced by a pair of T-posts with 25.4 cm spacing towards the entrance of the WE along the right-of-way and narrowing down to 10.2 cm at the structure’s exit on the non-road side of the fence. Furthermore, the opening above the mount was extended another 38 cm and its body was covered with a 1.3 × 1.3 cm mesh wire extending about 38 cm from the entrance of the fence to an opening at the habitat side. Of 10 WEs in Design B, six had a one-way wooden frame door with transparent plexiglass panels secured with a spring-loaded hinge at the joining of the wooden frame. This design with the door was intended to allow animals from the road or ROW side to open the door by pushing it but would prevent animals from opening it from the habitat side of the fence. Moreover, three major changes were made in Design B compared to Design A (1) installation of the raised berm, (2) shortening and narrowing the funnel, and (3) covering the structure with finer size mesh wire. Unlike the WEs in Design B, the modified WEs in Design C shared the same structural features as Design B except for the raised berm, which was removed from all sites.

### Data collection

Monitoring of Design A began in June 2019 and ended in November 2020, with varied monitoring days for each WE site (S1 Table). Monitoring of Design B occurred from November 2021 to February 2023 with four sites starting in November and December and the other six sites starting from January 2022. The monitoring of Design C began in May 2023 and ended in June 2024. There was a discrepancy in the monitoring period between WE sites because some of the sites in Design A were closed early in response to the coyote and bobcats using the WEs in an unintended direction. In order to account for the variation in monitoring days between each WE sites, the number of days was trimmed to an average of 335 ± 27 days for each study period.

**Fig 3:**
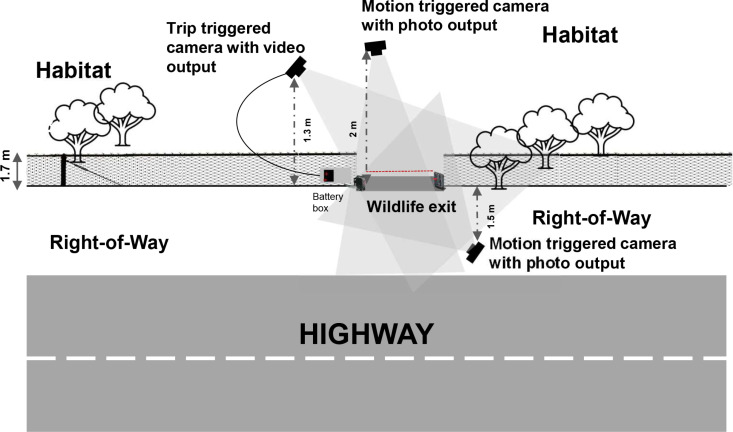
Illustration of camera trap position at wildlife exit sites along State Highway 100, Cameron County, Texas. Two cameras were positioned at the habitat side of the fenced roadway (a trip-triggered camera captures 30-second video and a motion-trigger camera captures three images per trigger, and one motion-triggered camera at the right-of-way with three photo outputs per trigger. All three cameras were faced toward the entrance of the wildlife exit to capture the interactions of wildlife with the structure.

Wildlife interactions with the WE at each site were recorded using two different types of trigger methods of camera traps: active infrared (AIR) and passive infrared (PIR). Design A had a pair of PIR cameras (HyperFire2, Reconyx, USA) deployed with one camera installed at the habitat side and another on the ROW or roadside of the fence, both facing toward the WE structure (Fig 3). These were motion triggered cameras programmed to take three images within a second interval difference. For Designs B and C, the third AIR-triggered camera, an external infrared trip wire system (Seco-Larm E-931S35RRQ13 Enforced Indoor/Outdoor Wall Mounted Photoelectric Beam Sensor, Seco-Larm, USA), was installed at all 10 WE sites, programmed to take a 30-second video. This was added to account for the restricted field of view due to the addition of the berm and doors. All three cameras installed at each WE site were faced towards the entrance of the structure and placed at the average distance of 1.6 m from the fence (Fig 3).

Four different environmental variables along with design period (Design A, B, C with door and no door) were tested as factors: distance to the nearest WG, distance to the nearest WCS, canopy cover, and temperature. The distance to the nearest mitigation measures (WG and WCS) was determined by using the Euclidean distance tool in ArcGIS Pro to see if distance to these mitigation measures was a factor influencing meso-carnivore use of WEs. This factor was considered based on the assumption that the distance to the nearest WG and WCS could increase or decrease the frequency of WE use. The nearest distance to WG was categorized into six categories (S2 Table). Similarly, the distance to the nearest WCSs was also categorized into six categories (S2 Table). The canopy cover of the vegetation in WE sites was measured using a densitometer, which was categorized into three categories (S2 Table) because results from Lombardi et al. [17] suggest a strong linkage between ocelot occurrence and dense canopy cover. The daily temperature data for each monitoring period was collected from the Laguna Atascosa weather station (Station ID: ATRT2; WIMS ID: 418603; Approximately 16 km north of Laguna Vista) [19] and categorized into five categories (S2 Table). Temperature was used as a factor because it can influence species geographic ranges, metabolic rates and physiological stress.

### Data management

**Fig 4:**
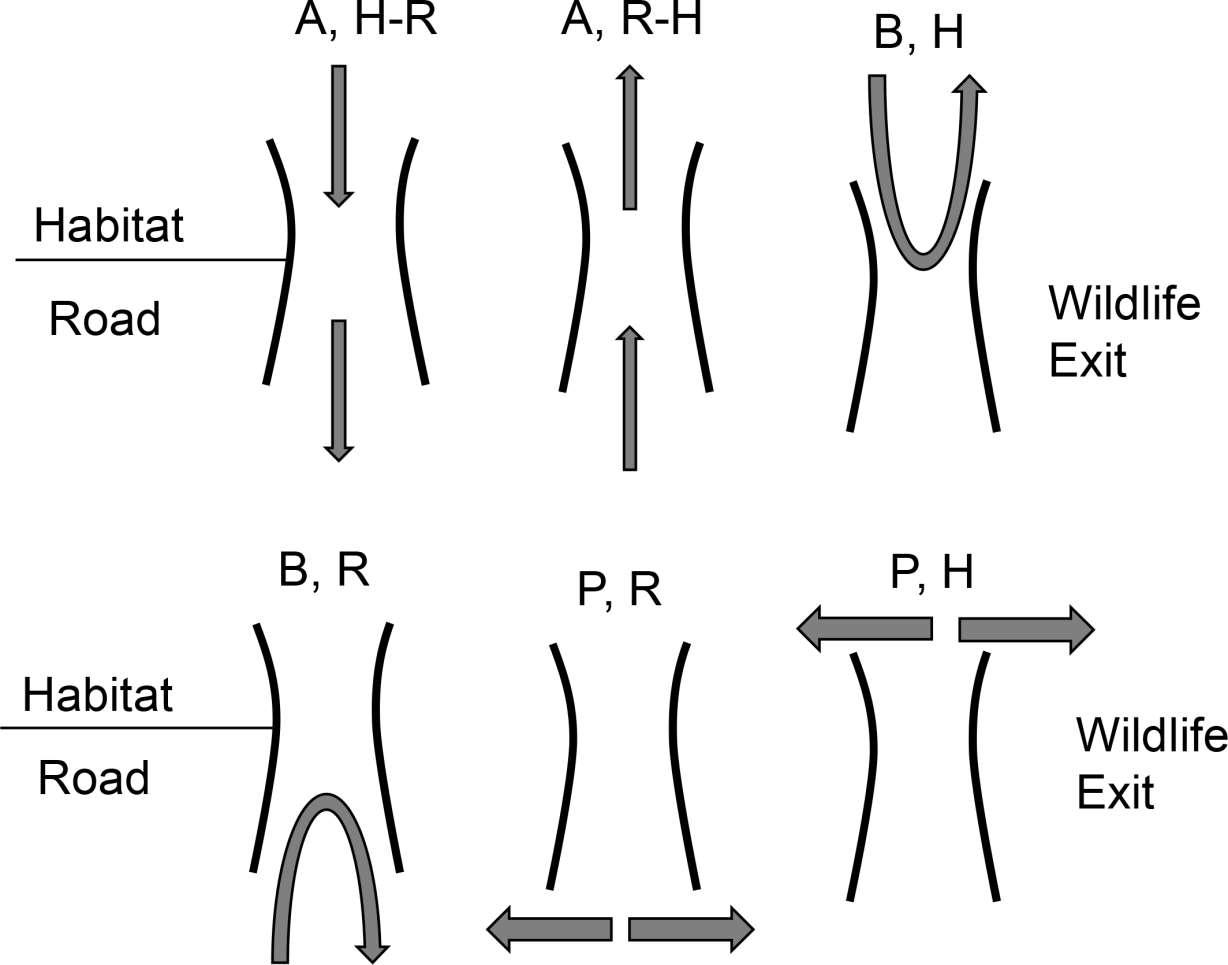
Wildlife interaction categorization at wildlife exit on State Highway 100, Cameron County Texas. Class A: Interactions of individuals making successful crossings from habitat to road (H-R) or road to habitat (R-H). Class B: Interactions where entry/exit occurs on the same side of the chain-link fence (Habitat: H, Road: R) and Class P: Interactions where individuals move parallel to the fence either in road (R) or habitat (H) without crossing the wildlife exit.

All the cameras at each WE site were checked bi-weekly to collect data and ascertain that cameras and trip sensors were working efficiently. All the field data (images and videos) obtained from each WE site were relabeled based on their timestamps using the ReNamer program (version: 6.8.0.0, Den4b Team) and sorted into folders based on species and then by the number of individuals. To quantify wildlife behavior at the WEs, four different behavioral classes were used to classify behavior. Class A indicates the successful crossing of WE from either side of the fence. This class was further divided into sub-classes to indicate wildlife crossings at the WEs: R-H for road to habitat and H-R for habitat to road. Class B indicates wildlife entering the WE but does not cross it completely and returns from the same side of the structure. Similarly, class P indicates that an individual is not interacting with the WE but moving parallel to the fence (Fig 4).

### Data analysis

The analysis of the WE data was conducted using Microsoft Excel and R Studio (version 2024.04.1 + 784), with all significant testing performed at an alpha value of 0.05 based on “Approach” and “No Approach”. These metrics were used instead of using crossing rate because the overall purpose of the new WE design was to see if those structures would be able to attract more meso-carnivore individuals from the roadside of the fence toward the structures. To determine if sites with a door (referred to as “door”) and without a door (referred to as “no door”) were contributing factors influencing the use of these WEs by meso-carnivores, WE sites in Design A where a door was installed since the start of Design B were categorized as “Design A: Future Door” and sites where the door was not installed as “Design A: No Future Door”. Similarly, WEs in Design B with a door and raised berm were categorized as “Design B: Door” and without the door as “Design B: No Door”. Lastly, raised berm from all WE sites were removed in Design C, which was categorized as “Design C: Door” and “Design C: No Door” (Fig 2).

A Generalized Linear Model (GLM) with logit function was used as a statistical modelling tool to analyze the WE categories based on the direction of targeted species movement (H-R and R-H) by keeping canopy cover, species, distance to the mitigation measures (WCS and WG), temperature, and design types (Design A, B, and C) as factors. This statistical method was adopted due to its flexibility with the distribution curve as data does not necessarily have to be normally distributed, unlike traditional linear regression [20]. In addition, our data was expressed in binary form (either true = 1 or false = 0), where GLM can accommodate response variables that follow a binary distribution, which ensures that predicted probabilities lie between 0 and 1 [21]. For the analysis, we coded 1 for an approach interaction (Class: A and B) and 0 for behavior in which wildlife did not interact with the WE (Class P). The multicollinearity of the GLM, having at least two factors, was checked using the Variance Inflation Factor (Vif) function in the R package “car” [22] to measure the degree of multicollinearity among predictors in a regression model. A higher Vif value indicates that predictors may be highly collinear with the other predictors, which could reduce the stability and reliability of the regression coefficient estimates in the model [23]. So, predictors with higher multicollinearity were removed from the model. The overdispersion was also checked by dividing the Pearson residuals with residual degrees of freedom to visualize how well the selected GLM fits the data [24]. ANOVA with type III was used to see if the variation in the dependent variable (response variable) could be explained by the predictor variables and if these differences were statistically significant. A pairwise comparison was done using “emmeans” (R package) Tukey test to examine the contrasts between different levels in the factors [25].

### Ethics statement

Ethical approval was not required for the study involving animals in accordance with the local legislation and institutional requirements because the data collected in this study was observational camera trap data only. This research was conducted within the defined right-of-way of SH 100; therefore, no permits were necessary.

## Results

**Fig 5:**
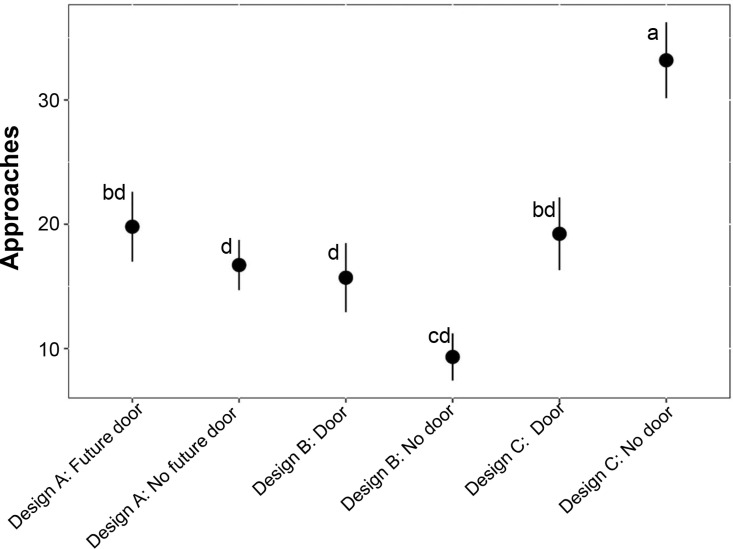
The mean approaches of four meso-carnivores (bobcat, coyote, northern raccoon, and striped skunk) at the wildlife exits along the State Highway 100, Cameron County, Texas, analyzed from the roadside of the fence based on design and door types. The approaches were categorized into six groups (Design A: Future Door and Design A: No Future Door; Design B: Door and Design B: No Door; Design C: Door and Design C: No Door). It was determined based on approach interactions (Class A and B) with directions from road to habitat (R-H) and road (R). Letters indicate pair-wise comparisons with significant values (P < 0.05) with bar indicating a standard error (SE).

**Fig 6:**
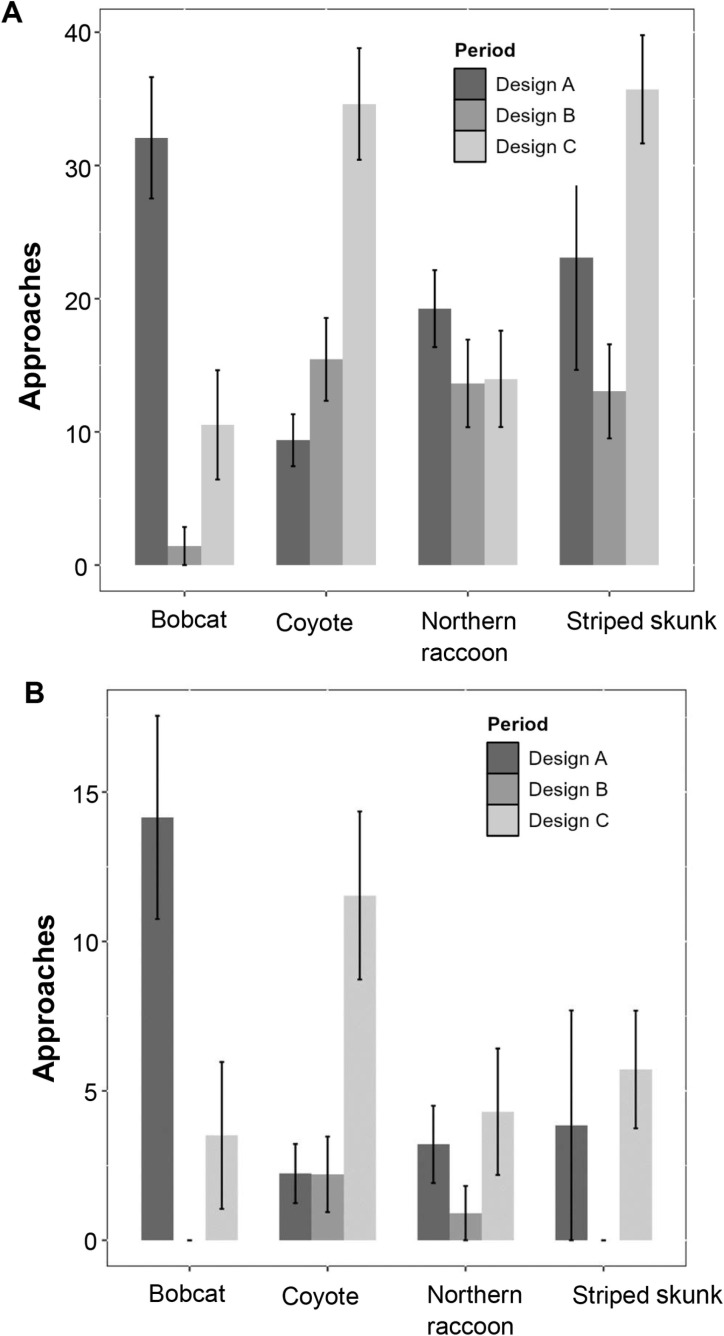
Mean approaches of four meso-carnivore species (bobcat, coyote, northern raccoon, and striped skunk) at wildlife exits on State Highway 100 in Cameron County, Texas, analyzed based on three wildlife exit design types (Design A, B, and C) with bar indicating as standard error (SE). Figure A illustrates the mean approaches of species on the “Road” side and Figure B shows the mean approaches from the “Habitat” side of the fence. Interactions classified as Class A and B were considered “Approach” while interactions from Class P as “No Approach”.

**Fig 7:**
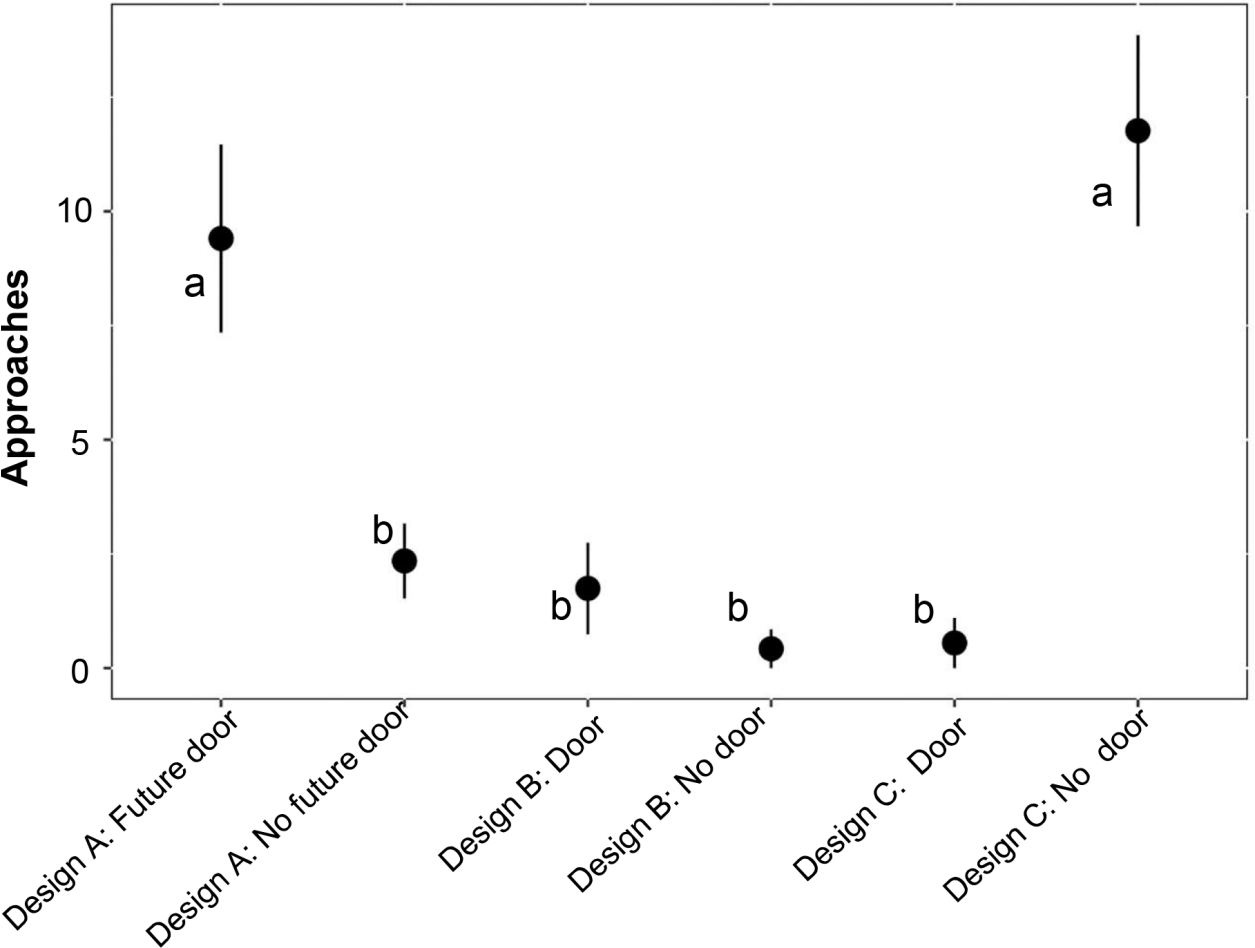
The mean approaches of four meso-carnivores (bobcat, coyote, northern raccoon, and striped skunk) at the wildlife exits along the State Highway 100, Cameron County, Texas, analyzed from the habitat side of the fence based on design and door types. The approaches were categorized into six groups: Design A: Future Door; Design A: No Future Door; Design B: Door; Design B: No Door; Design C: Door; Design C: No Door). It was determined based on approach interactions (Class: A and B) with directions from habitat to road (H-R) and habitat (H). Letters indicate pair-wise comparisons with significant values (P < 0.05) and bars indicating standard error (SE).

A total of 14,120 mammalian interactions across 19 taxa were recorded during the study period for Design A, B, and C. In Design A, there were 6,561 interactions, of which 545 involved the four target species (striped skunk, bobcat, northern raccoon, and coyote). Design B recorded 3,601 interactions, with 440 from the target species, while Design C had 3,958 interactions, including 424 from the target species (S3 Table). The mean approaches of all four target species from the R-H were 17.86 ± 0.39 in Design A, 12.01 ± 0.46 in Design B, and 27.14 ± 0.42 in Design C. Among those three designs, 19.80 ± 0.63 approaches were observed in Design A with future door, 16.72 ± 0.49 in Design A with no future door, 15.70 ± 0.70 in Design B with door, 9.32 ± 0.62 in Design B with no door, and 19.23 ± 0.67 in Design C with door and 33.19 ± 0.53 in Design C with no door (Fig 5). Additionally, 32.08 ± 0.80 approaches were recorded for bobcat in Design A with the door (0.76) and no door (0.62), 1.43 ± 1.20 in Design B (Door: 0.5; No Door: 0), and 10.53 ± 1.26 in Design C (Door: 0; No Door: 0.86; Table 1) whereas, the mean approaches for coyote from road side of the fence were 9.38 ± 0.64 in Design A, 15.44 ± 0.79 in Design B, and 34.62 ± 0.71 in Design C (Fig 6A). Similarly, the mean approaches of the four target species from H-R were 4.97 ± 0.42 in Design A (No future door: 2.35 ± 0.54; future door: 9.41 ± 0.67), 0.98 ± 0.49 in Design B (Door: 0.61 ± 0.78; No door: 0.42 ± 0.65), and 6.90 ± 0.47 in Design C (Door: 0.55 ± 0.74; No door: 11.76 ± 0.61; Fig 7). The mean approaches for bobcat were 14.15 ± 0.90 in Design A (Door: 0.34; No Door: 0.14) and 3.51 ± 1.31 in Design C (Door: 0; No Door: 0.12; Table 2), while no approach interactions were observed in Design B. For coyote, the mean approaches were 2.23 ± 0.66, 2.21 ± 0.85, and 11.54 ± 0.83 for Design A, B and C respectively (Fig 6B).

**Table 1:** Mean approaches of mammalian species from the roadside of the fence. The data involved four mammalian species at ten WE sites, analyzed across three WE designs (Design A, B, and C). Approaches were calculated based on the sites with “Door” and “No Door” categories along with interactions classified into “Approach” (Class A and B, direction R-H and R) and “No Approach” (Class P, direction R).

Design	Species	Design A	Design B	Design C
Door	Bobcat	0.76	0.50	0
	Coyote	0.50	0.34	0.53
	Northern raccoon	0.38	0.25	0.45
	Striped skunk	0	0.25	0.50
No Door	Bobcat	0.62	0	0.86
	Coyote	0.92	0.27	0.85
	Northern raccoon	0.60	0.32	0.44
	Striped skunk	0.86	0.44	0.87

**Table 2:** Mean approaches of mammalian species from the habitat side of the fence. The data involved four mammalian species at ten WE sites, analyzed across three WE designs (Design A, B, and C). Approaches were calculated based on the sites with “Door” and “No Door” categories along with interactions classified into “Approach” (Class A and B, direction H-R and H) and “No Approach” (Class P, direction H).

Design	Species	Design A	Design B	Design C
Door	Bobcat	0.34	0	0
	Coyote	0.13	0.09	0.03
	Northern raccoon	0.05	0.05	0
No Door	Bobcat	0.14	0	0.12
	Coyote	0	0.02	0.39
	Northern raccoon	0.06	0	0.11
	Striped skunk	0.10	0	0.15

The result from GLM testing the first hypothesis that the approaches of the target species increase from road to habitat (R-H) side in Design C compared to Design A, found that WE design (X^2 ^= 42.903, d.f. = 5, p < 0.001), canopy cover (X^2 ^= 4.530, d.f. = 1, p = 0.0333), and distance to nearest WG (X^2 ^= 20.776, d.f. = 4, p = 0.0003507) were significant predictors to the model (S4 Table). No multicollinearity was observed in the predictor of the model as all those variables were in the categorical form. Also, no over-dispersion (dispersion parameter = 1.004) was detected in the model using “The Pearson Chi-Square test for Overdispersion”. The mean approaches of all four target species from R-H in Design C (27.14 ± 0.42) were much higher compared to the mean approaches in Design A (17.86 ± 0.39). Furthermore, the pairwise Tukey test from “emmeans” package illustrated that there was a significant difference in approaches between Design A with future door (19.80 ± 0.63) – Design C with no door (33.19 ± 0.53; p = 0.0221), Design A with no future door (16.72 ± 0.49) – Design C with no door (p = 0.0001), and Design C with door (19.23 ± 0.67) – Design C with no door (p = 0.0199; Fig 5).

The GLM for testing the second hypothesis, removal of the raised berm at the entrance of the WE site significantly increases the approaches of target species from the R-H side of Design C with no door compared to the same sites of Design B, also found that WE design as a significant predictor to the model. Among the two different WE door types between Design B and C, the WE in Design C without a door (33.19 ± 0.53) had a higher mean approach compared to Design B without a door (9.32 ± 0.62). Furthermore, the pairwise Tukey test from “emmeans” package showed that there was a significant difference in approaches between Design B with no door (9.32 ± 0.62) – Design C with no door (33.19 ± 0.53; p < 0.001), Design B with no door (9.32 ± 0.62) – Design C with door (19.23 ± 0.67; p = 0.0469), and Design B with door (15.70 ± 0.70) – Design C with no door (p = 0.0013; Fig 5). However, no significant difference in mean approaches were observed between Design B (14.63 ± 0.72) and C with door (p = 0.9530; Fig 5).

## Discussion

The comparison of approaches between three WE designs produced varied responses from meso-carnivores. Our study indicates that the installation of raised berm (Design B), as a deterrent structure to prevent H-R movement, reduced the overall R-H approaches of meso-carnivores. This assumption was verified when the removal of the ramp as a deterrent structures (Design C) significantly increased approaches in the sites without the door. Although no significant difference was observed at WE sites with a door between Design B and C, a slight increase in meso-carnivores approaches were detected. Initially, WE design (Design A) was conceptualized and installed to provide an escape option for ocelots stranded within the ROW, towards the habitat side of the fence. However, no ocelots were detected within the entire monitoring period of our study probably because of their low population and crossing frequency around SH100. To assess its effectiveness, the approaches of four meso-carnivores (coyote, bobcat, northern raccoon, and striped skunk) were used.

The main goal of this study was to determine if the change in WE design would increase the corrective use of WE structure by the target species and potentially ocelots who might have entered the ROW through nearby mitigating structures. Our results show that the initial WE design (Design A) was effective in allowing small to medium-sized meso-carnivores to escape the ROW. However, the absence of a physical barrier failed to stop them from using the structure in an unintended direction [12]. This type of issue is quite common and has been documented in several studies [11,15,26]. Physical barriers placed at the top edge of the jump-outs [11] and fence gaps with one-way doors are the most common methods adopted to prevent those unintended movements. Although such methods have been shown to discourage large ungulates (elk, bighorn sheep, and deer) from using the mitigation measures in the wrong direction, the same techniques are less effective at deterring carnivores at wildlife jump outs [11]. A similar approach was tested in a previous study by adding a raised berm and door in Design B, which prevented the H-R movement of meso-carnivores at WE sites (Fig 7). Although effective in preventing unintended movements, this design discouraged overall meso-carnivores from using the structures altogether. Failure to use the crossings could be due to the difference in size and features of the deterrent structures that were used in our study. This could be due to the constricted wideness of the WE design and a wire mesh covering the structure, making it less attractive and crossable to the target species.

Our results support the hypothesis that the approaches of meso-carnivores increase significantly in Design C compared to Design A at WE sites without a door. The increased R-H approaches suggest that the target species became more familiar with the WE and approached it more frequently over time. Similar observations have been reported in bears [27], ungulates [28], and other mammals [29] using mitigation structures, where target species initially hesitated to use the mitigation measure before starting to use it frequently. Although the presence of a door at the WE site did not result in a significant change in the approaches compared to the same sites without a door in Design A, a slight decrease in the approaches was observed suggesting that the door was a potential factor in decreasing the approaches of meso-carnivores.

Our hypothesis that the installation of a raised berm in Design B would decrease the approaches of meso-carnivores was also confirmed. To verify if the raised berm was the major cause for the decrease in the R-H approaches, a comparison was made between Design B and Design C, which showed a significant increase in the approach once the berm was removed at the sites without the door. Although no significant difference was seen between Design B and C with a door, a slight increase in approaches was observed when the raised berms were removed. This indicates the installation of a raised berm at the WE site is the major factor influencing the approach behavior of meso-carnivores. The approach behavior was demonstrated in the related study where the dimension and size of the crossing structure are interlinked where larger animals are more attracted towards wider and larger structures than compared to smaller animals [30].

Prior research suggests that carnivore species prefer some form of cover around and over the mitigation structure [7] so approaches of meso-carnivores could be influenced by the presence of canopy cover. Although no significant difference between the approaches of meso-carnivores between canopy covers was observed, slightly higher approaches were detected in the sites with mixed canopy cover (19.69 ± 0.32) than compared to sites without any cover (18.06 ± 0.37). This small difference in approaches might be due to the higher number of WE sites with open covers (n = 6) than the mixed covers (n = 4). The activity of bobcats in our study were slightly higher in the WE sites with mixed canopy covers. Similar behavior was seen in the study done by Sheikh et al. [12] and Horne et al. [31], where bobcat in South Texas preferred habitat with open to mix canopy covers. Ocelots on the other hand prefer areas with dense vegetation and canopy covers [17,31,32], which would likely benefit from the WE constructed in dense vegetation habitats along the section of SH100.

Studies have shown that areas with abundant food, water sources, and cover could be the major factors supporting carnivore populations, such as bobcat, coyote, and ocelots [33, 34, 35, 36]. Anthropogenic activity could also influence the approaches of the target species because the WE present at the ROW have limited cover availability with only grasses present, which often get mowed leaving bare strips of land without any bushes and trees for wildlife to hunt for prey or escape from predators.

Among the four target species, the frequency and approaches of coyotes were the highest in all three WE design types. Among the coyotes that approached from the roadside, several attempts were made before successfully making R-H crossing in both the “Door” and “No Door” sites. Similar findings have been reported in the study carried out by Sheikh et al. [12] on SH100, where coyotes managed to cross the structure after struggling for a while to fit through the WE. Compared to the study by Sheikh et al. [12] with Design A, bobcat interactions were much lower after the installation of the berm and door in Design B. However, interactions slightly increased again after the berm was removed later in Design C, in the R-H direction. The reduction in the approaches could also be attributed to various changes made within the monitoring period such as controlled burning done for Aplomado falcon (*Falco femoralis*) habitat restoration, periodic mowing of grasses within the SH 100 ROW complex, and various environmental changes like drought, high temperature, and heavy rainfall that might have occurred within the study period.

The approaches of meso-carnivores from R-H were much higher than compared to the H-R in WE with Design C. Although higher approaches were observed, the number of species detected at the habitat side was much greater. However, the use of the WE was limited due to barriers such as the door and raised berm, providing evidence that the size and shape of mitigation measures impact its effectiveness [37]. This could indicate that the fence has been an effective barrier in preventing wildlife from accessing the roadway and that the WE have been effective in attracting more meso-carnivores from the roadside than in repelling them.

## Conclusion

The WE study (Design C) was carried out to assess its effectiveness compared to the previous two WE designs (i.e., Design A and B). Our study shows significant connection between the raised berm in Design B and a decline in the “Approaches” of meso-carnivore communities toward the WE sites. With the berm removal in Design C, the approaches of target species from R-H increased significantly in the sites without a door. Although a slight increase in “Approaches” was observed, no significant change between the two WE designs (B and C) with a door was detected. Moreover, it was expected that target species would eventually learn to use WE sites over time which was observed from this study as more R-H approaches were recorded in Design C, sites with no door, than the same sites in Design A. Our study also indicates that the WE door design might be another structural cause for limiting its effectiveness, where significantly lower approaches were recorded for meso-carnivores in sites with a door compared to the sites with no door in Design C.

Findings from this study can be a valuable tool for designing new WEs that can promote higher R-H movement while preventing H-R movement, especially for meso-carnivore species. Based on the preliminary results, a new WE design was installed in all ten WE sites with a one-way door and bars that can move independently. This design was inspired by the pigeon door having a lightweight independent steel bar that can be opened by pushing it from one side and closes itself due to gravity once the force is lifted. While this design is novel from any other published design, its effectiveness is yet to be assessed. The wider body of this door design not only makes it effective for small to medium-sized carnivores but also for medium-size ungulate like javelina and feral hogs.

## Supporting information

S1 Table Monitoring periods for wildlife exits (WE) under Design A, B, and C. To avoid bias, the same number of days were chosen for each WE site across the three different design periods. In Design A, the start date was the day monitoring began, and the end date was the day monitoring ended. The number of monitoring days varied among WE sites in Design A due to their different start and end dates. For Design B and Design C, the number of monitoring days for each WE site was standardized based on the corresponding WE site days from Design A.
(DOCX)

S2 Table Category Definitions. A) Categories defined by distance, B) defined by canopy cover, and C) defined by temperature on SH 100.
(DOCX)

S3 Table Species detections at wildlife exits. Meso-carnivore species observation detected in SH 100 based on design type (Design A, B, C), door design (Door and No Door), and direction (Road to Habitat: R-H, Road: R, Habitat to Road: H-R, Habitat: H).
(DOCX)

S4 Table GLM outputs. Generalized Linear Model (GLM) outputs summary showing coefficients, odds ratio and analysis of deviance for all the variables (Design type, Species, Temperature, Distance to nearest wildlife guard – WG, Distance to nearest wildlife crossing structure – WCS, Canopy cover) from road to habitat (R-H) interactions.
(DOCX)
