# Supplementary material for: Assessing the effects of design modifications on the use of wildlife exits designed for endangered Texas ocelots
Source: PLoS One. 2025 Jun 24;20(6):e0323705. doi: 10.1371/journal.pone.0323705 (PMC12186924; doi:10.1371/journal.pone.0323705)
Supplement: S1 Table — To avoid bias, the same number of days were chosen for each WE site across the three different design periods. In Design A, the start date was the day monitoring began, and the end date was the day monitoring ended. The number of monitoring days varied among WE sites in Design A due to their different start and end dates. For Design B and Design C, the number of monitoring days for each WE site was standardized based on the corresponding WE site days from Design A. (DOCX) [file pone.0323705.s001.docx]

**S1 Table. Monitoring periods for wildlife exits (WE) under Design A, B, and C.** To avoid bias, the same number of days were chosen for each WE site across the three different design periods. In Design A, the start date was the day monitoring began, and the end date was the day monitoring ended. The number of monitoring days varied among WE sites in Design A due to their different start and end dates. For Design B and Design C, the number of monitoring days for each WE site was standardized based on the corresponding WE site days from Design A.

| Site | Design A Date Range | | Days | Design B Date Range | | Days | Design C Date Range | | Days |
| --- | --- | --- | --- | --- | --- | --- | --- | --- | --- |
|  | Start | End |  | Start | End |  | Start | End |  |
| WE01 | 10/18/2019 | 11/13/2020 | 392 | 1/6/2022 | 2/2/2023 | 392 | 5/10/2023 | 6/5/2024 | 392 |
| WE02 | 10/18/2019 | 11/13/2020 | 392 | 1/6/2022 | 2/2/2023 | 392 | 5/10/2023 | 6/5/2024 | 392 |
| WE03 | 10/18/2019 | 11/13/2020 | 392 | 11/25/2021 | 12/22/2022 | 392 | 5/10/2023 | 6/5/2024 | 392 |
| WE04 | 10/18/2019 | 7/17/2020 | 273 | 1/6/2022 | 10/6/2022 | 273 | 5/10/2023 | 2/7/2024 | 273 |
| WE05 | 6/25/2019 | 5/31/2020 | 341 | 11/24/2021 | 10/31/2022 | 341 | 5/10/2023 | 4/15/2024 | 341 |
| WE06 | 6/25/2019 | 7/17/2020 | 388 | 12/1/2021 | 12/24/2022 | 388 | 5/10/2023 | 6/1/2024 | 388 |
| WE07 | 10/18/2019 | 11/13/2020 | 392 | 12/1/2021 | 12/28/2022 | 392 | 5/10/2023 | 6/5/2024 | 392 |
| WE08 | 10/18/2019 | 11/13/2020 | 392 | 1/6/2022 | 2/2/2023 | 392 | 5/10/2023 | 6/5/2024 | 392 |
| WE09 | 10/18/2019 | 5/12/2020 | 207 | 1/6/2022 | 8/1/2022 | 207 | 5/10/2023 | 12/3/2023 | 207 |
| WE10 | 10/18/2019 | 4/13/2020 | 178 | 1/6/2022 | 7/3/2022 | 178 | 5/10/2023 | 11/4/2023 | 178 |
| Mean (SE) |  |  | 335 (27) |  |  | 335 (27) |  |  | 335 (27) |
