# Supplementary material for: Assessing the effects of design modifications on the use of wildlife exits designed for endangered Texas ocelots
Source: PLoS One. 2025 Jun 24;20(6):e0323705. doi: 10.1371/journal.pone.0323705 (PMC12186924; doi:10.1371/journal.pone.0323705)
Supplement: S2 Table — A) Categories defined by distance, B) defined by canopy cover, and C) defined by temperature on SH 100. (DOCX) [file pone.0323705.s002.docx]

**S2 Table. Category Definitions.** A) Categories defined by distance, B) defined by canopy cover, and C) defined by temperature on SH 100.

A. Categories defined for distance to wildlife guards (WGs) and distance to wildlife crossing structures (WCSs) on SH 100.

| Category | Distance to WG (in meter) | Distance to WCS (in meter) |
| --- | --- | --- |
| Very close | 0 – 50 | 0 – 100 |
| Close | 51 - 100 | 101 – 500 |
| Moderate | 101 – 200 | 501 – 1000 |
| Far | 201 – 500 | 1001 – 2000 |
| Very far | 501 – 1000 | 2001 – 3000 |
| Extremely far | >1000 | >3000 |

B. Categories defined for canopy cover (%) on SH 100.

| Category | Canopy (%) |
| --- | --- |
| Open | 0 – 30 |
| Mixed | 31 – 70 |

C. Categories defined for temperature on SH 100.

| Category | Degree Celsius |
| --- | --- |
| Freezing | -4 - 0^o^C |
| Cold | > 0 – 10^o^C |
| Moderate | > 10 – 20^o^C |
| Warm | > 20 – 30^o^C |
| Hot | > 30^o^C |
