# Supplementary material for: Assessing the effects of design modifications on the use of wildlife exits designed for endangered Texas ocelots
Source: PLoS One. 2025 Jun 24;20(6):e0323705. doi: 10.1371/journal.pone.0323705 (PMC12186924; doi:10.1371/journal.pone.0323705)
Supplement: S3 Table — Meso-carnivore species observation detected in SH 100 based on design type (Design A, B, C), door design (Door and No Door), and direction (Road to Habitat: R-H, Road: R, Habitat to Road: H-R, Habitat: H). (DOCX) [file pone.0323705.s003.docx]

**S3 Table. Species detections at wildlife exits.** Meso-carnivore species observation detected in SH 100 based on design type (Design A, B, C), door design (Door and No Door), and direction (Road to Habitat: R-H, Road: R, Habitat to Road: H-R, Habitat: H).

| Design | Species | Design A | Design B | Design C |
| --- | --- | --- | --- | --- |
| R-H and R | | | | |
| Door | Bobcat | 21 | 2 | 0 |
|  | Coyote | 18 | 41 | 32 |
|  | Northern raccoon | 40 | 32 | 19 |
|  | Striped skunk | 1 | 12 | 18 |
| No Door | Bobcat | 29 | 4 | 6 |
|  | Coyote | 13 | 26 | 33 |
|  | Northern raccoon | 35 | 18 | 9 |
|  | Striped skunk | 7 | 18 | 47 |
| H-R and H | | | | |
| Door | Bobcat | 35 | 16 | 35 |
|  | Coyote | 38 | 23 | 29 |
|  | Northern raccoon | 40 | 21 | 28 |
|  | Striped skunk | 9 | 25 | 21 |
| No Door | Bobcat | 21 | 48 | 16 |
|  | Coyote | 155 | 46 | 36 |
|  | Northern raccoon | 72 | 39 | 37 |
|  | Striped skunk | 9 | 37 | 54 |
