# Supplementary material for: Assessing the effects of design modifications on the use of wildlife exits designed for endangered Texas ocelots
Source: PLoS One. 2025 Jun 24;20(6):e0323705. doi: 10.1371/journal.pone.0323705 (PMC12186924; doi:10.1371/journal.pone.0323705)
Supplement: S4 Table — Generalized Linear Model (GLM) outputs summary showing coefficients, odds ratio and analysis of deviance for all the variables (Design type, Species, Temperature, Distance to nearest wildlife guard – WG, Distance to nearest wildlife crossing structure – WCS, Canopy cover) from road to habitat (R-H) interactions. (DOCX) [file pone.0323705.s004.docx]

**S4 Table. GLM outputs**. Generalized Linear Model (GLM) outputs summary showing coefficients, odds ratio and analysis of deviance for all the variables (Design type, Species, Temperature, Distance to nearest wildlife guard - WG, Distance to nearest wildlife crossing structure - WCS, Canopy cover) from road to habitat (R-H) interactions.

GLM summary

|  | Variables | Estimate | Std. Error | z value | Pr(>\|z\|) | Odd ratio |
| --- | --- | --- | --- | --- | --- | --- |
|  | (Intercept) | -3.2289 | 64.94896 | -0.05 | 0.96035 | 0.0396012 |
| Period Design | DesignA.No.Future.Door | 0.15063 | 0.21189 | 0.711 | 0.47716 | 1.1625693 |
|  | DesignB.Future.Door | 0.91792 | 0.18677 | 4.915 | 0.000000889*** | 2.504073 |
|  | DesignB.No.Door | 0.0118 | 0.31642 | 0.037 | 0.97025 | 1.0118716 |
|  | DesignC.Door | -0.28941 | 0.19653 | -1.473 | 0.14085 | 0.7487067 |
|  | DesignC.No.Door | -0.87984 | 0.43485 | -2.023 | 0.04304* | 0.4148493 |
| Species | Coyote | 0.02476 | 0.15396 | 0.161 | 0.87224 | 1.0250688 |
|  | Northern.Raccoon | -0.05838 | 0.12038 | -0.485 | 0.62773 | 0.9432952 |
|  | Striped.Skunk | -0.20934 | 0.12773 | -1.639 | 0.10122 | 0.8111207 |
|  | Mixed | 0.81429 | 0.38327 | 2.125 | 0.03362* | 2.2575724 |
|  | Hot | -6.61783 | 205.3861 | -0.032 | 0.9743 | 0.0013363 |
| Temperature | Moderate | -5.33483 | 173.583 | -0.031 | 0.97548 | 0.0048208 |
|  | Cold | -2.98002 | 102.6937 | -0.029 | 0.97685 | 0.0507919 |
|  | Freezing | -1.35829 | 38.81549 | -0.035 | 0.97208 | 0.2570992 |
| Distance to nearest WCS | Extremely.Far | 0.3421 | 0.29553 | 1.158 | 0.24703 | 1.4079032 |
|  | Close | 0.13356 | 0.20739 | 0.644 | 0.51957 | 1.1428915 |
|  | Moderate | 0.57652 | 0.43811 | 1.316 | 0.1882 | 1.7798307 |
|  | Very.Far | 1.88629 | 0.69197 | 2.726 | 0.00641** | 6.5948242 |
| Distance to nearest WG | Extremely.Far | -0.40437 | 0.36445 | -1.11 | 0.26719 | 0.6673941 |

Analysis of deviance

| Variables | LR Chisq | Df | Pr(>Chisq) |
| --- | --- | --- | --- |
| Period.Design | 42.903 | 5 | 3.866e-08******* |
| Species | 4.591 | 3 | 0.2042836 |
| Canopy.Cover | 4.530 | 1 | 0.0333138***** |
| Temperature | 2.581 | 4 | 0.6302251 |
| Distance to nearest WCS | 1.367 | 1 | 0.2423686 |
| Distance to nearest WG | 20.776 | 4 | 0.0003507******* |
